# Closed Negative Suction Drain Entrapment in Total Knee Arthroplasty: A Report on the Implications of a Broken Drain Based on the ChatGPT Outlook

**DOI:** 10.7759/cureus.36290

**Published:** 2023-03-17

**Authors:** Lavindra Tomar, Gaurav Govil, Pawan Dhawan

**Affiliations:** 1 Department of Orthopedics, Max Super Speciality Hospital, Patparganj, Delhi, IND

**Keywords:** chat gpt, complication, entrapment, closed drain, suction drain, knee, total knee replacement, arthroplasty

## Abstract

Total knee arthroplasty (TKA) has been the gold standard treatment for end stage arthritis of knee. The advancements in techniques have allowed successful outcomes. The use of closed negative suction drain in TKA has been controversial. Entrapment of a drain following TKA with a broken drain has been reported rarely though it has important implications.

An obese 65-year-old female presented with painful bilateral knees. A clinic-radiological assessment confirmed an advanced grade osteoarthritis (OA). A single stage bilateral TKA was done. The use of closed negative suction drain for both knees was done as a routine protocol. The left knee drain got entrapped and an inadvertent pull due to abnormal positioning in flexed knee impinged and broke the drain. The right knee removal of drain on the second post-operative day was uneventful. A radiological assessment confirmed the position of the broken drain in left knee. A mini arthrotomy ensued with removal of the drain piece. Subsequent post-operative period was uneventful. The knee function recovered with painless full range of motion. There was no evidence of infection or implant loosening noted at a two-year follow-up.

The generative text model ChatGPT (OpenAI, USA) was used to identify the implication with the use of drains in TKA. The use of drains remains controversial with no clear consensus on its regular use. The breakage of drain poses an immediate concern with need for wound revision and removal of foreign body. The long-term observation for any knee infection, stiffness or poor knee function needs monitoring. The early identification can prevent late symptomatology.

The closed negative suction drain in our practice has become selective and presently has an infrequent use in TKA. An entrapment of a closed negative suction drain merits urgent measures. The remedial measures may ensure to preserve the knee joint function and maintain the ability to do activities of daily living.

## Introduction

Total knee arthroplasty (TKA) usually has successful outcomes in the end stage arthritis of the knee. The techniques have evolved to avoid complications and allow a near normal full recovery. The use of drains in TKA has been a topic of controversy without any consensus on its regular use [1,2].

The use of drains in TKA has certain increased risks. There are reports of increased risk for retrograde infection, more blood loss, delayed rehabilitation, and rarely drain breakage [3,4]. The post-operative occurrence of drain breakage in TKA may require wound revision and drain removal [5]. 

We present a report of a closed negative suction drain inadvertent forceful pull-off presenting with an entrapped broken drain piece. An immediate and early radiological identification allowed management by remedial measures such as a mini arthrotomy, exploration, and wound revision for the complete extraction of broken drain pieces. We used the artificial intelligence (AI) based generative text model ChatGPT (OpenAI, USA) to assess the implications of drain usage by posing multiple questions related to its safety, incidence, complications, etc. and noted the ChatGPT responses. 

## Case presentation

An obese 65-year-old female with a body mass index of 30 presented with persistent pain in both her knees since almost two years. There has been a marked inability to do activities of daily living without pain and support since around three months. Examination revealed painful flexion of both the knees. Gait was antalgic and there was markedly painful and restricted range of movements for bilateral knees. The markers for inflammation or infection including C-reactive protein, total white cell count, erythrocyte sedimentation rate were within normal ranges. Radiograph of both knees presented advanced osteoarthritic degeneration of bilateral knees. It was graded as Kellgren-Lawrence classification grade IV osteoarthritis (OA). 

**Figure 1:**
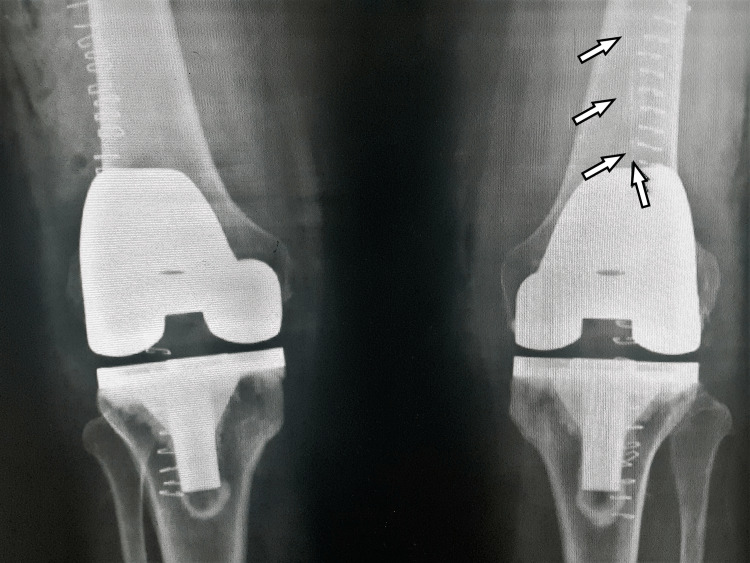
Post-operative radiograph of bilateral knees (anteroposterior view) Post-operative radiograph of bilateral knees in anteroposterior view with a broken drain in left knee marked with white arrows.

**Figure 2:**
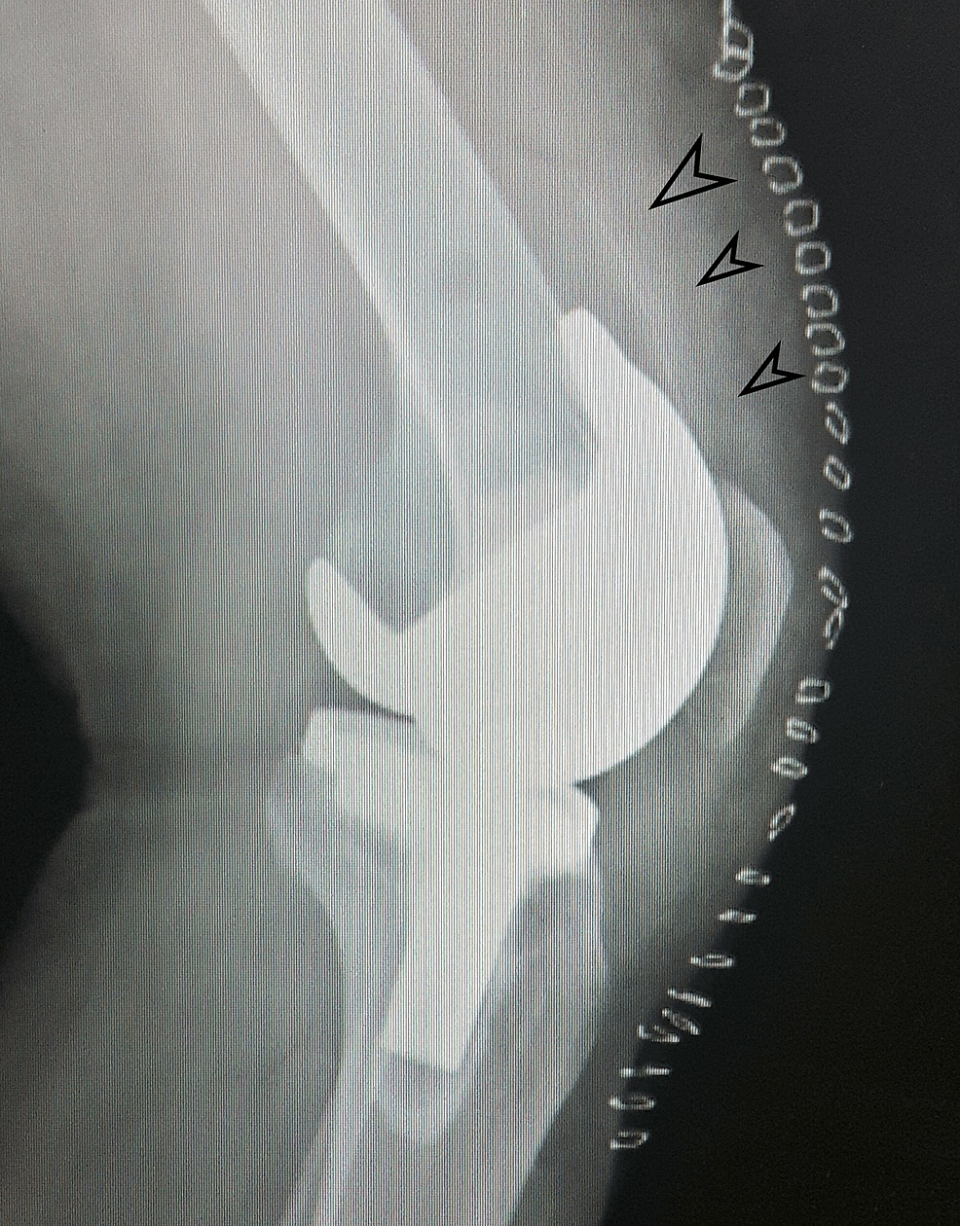
Post-operative radiograph of the left knee (lateral view) Post-operative radiograph of the left knee in lateral view delineating the broken drain piece with multiple black arrows.

Surgical management was done by single stage bilateral TKA. A closed negative suction drain (Romo-vac suction drain, No16; Romsons, India) was used in both the knees post-operatively. The drain was placed in the lateral gutter with a superolateral exit from the midline incision. The marker on the drain was ensured to be kept outside the skin margin. After completing the fascial closure with knee in flexion, the drain movement was checked for with knee in extension and observed to have a free smooth glide. The routine preventive measures were taken to ensure that the drain was free from any impingement under direct visualization before the complete wound closure. The drain was secured to the skin with a suture thereafter and a sterile dressing with compression bandaging was done. On the first post-operative day, the compression bandage was loosened out and the sterile dressing was changed. On the second post-operative day, the left knee closed suction drain got an inadvertent removal due to an abnormal forceful lateral positioning in bed resulting in pull out of the drain. A far lateral posture was given to patient with knee going into flexion for a routine dress change with indwelling drain left tied to the bed railing causing an inadvertent force and drain pull-out. An episode of acute severe knee discomfort was felt with subsequent painful straightening of the left lower limb. On examination of the pulled-out drain, there seemed a piece of drain which had broken off when observed for the drain in entirety. The initial soakage from the drain entry point was managed with sterile redressing and compression bandaging. The right knee negative suction drain was easily removed with a routine protocol by cutting the skin tagged suture and gently sliding out the drain. A radiological examination to identify a foreign body in the left knee was done immediately. The broken drain piece with a marker line was identified along the anterolateral aspect of the left knee radiograph in anteroposterior view (Figure 1) and a lateral view (Figure 2).

The femoral and tibial component were well-aligned in both the knees. The foreign body posed a significant concern, and an exploration surgery was planned.

**Figure 3:**
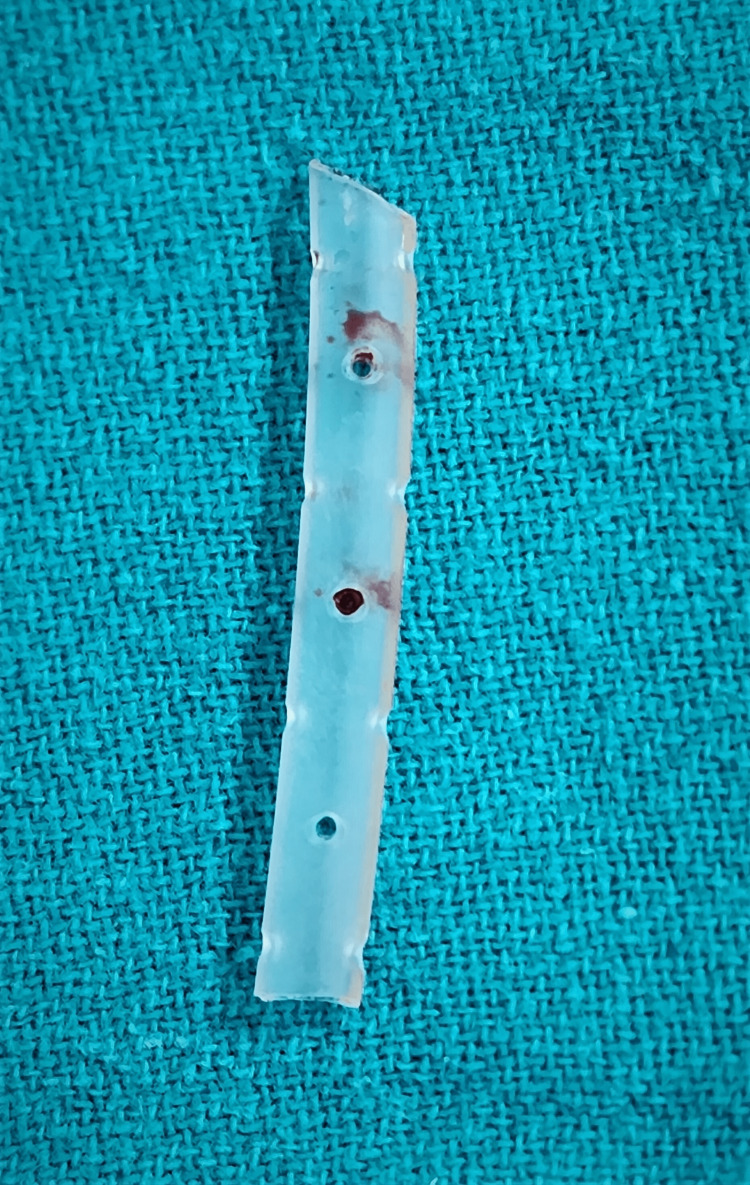
Per-operative image of the broken piece

**Figure 4:**
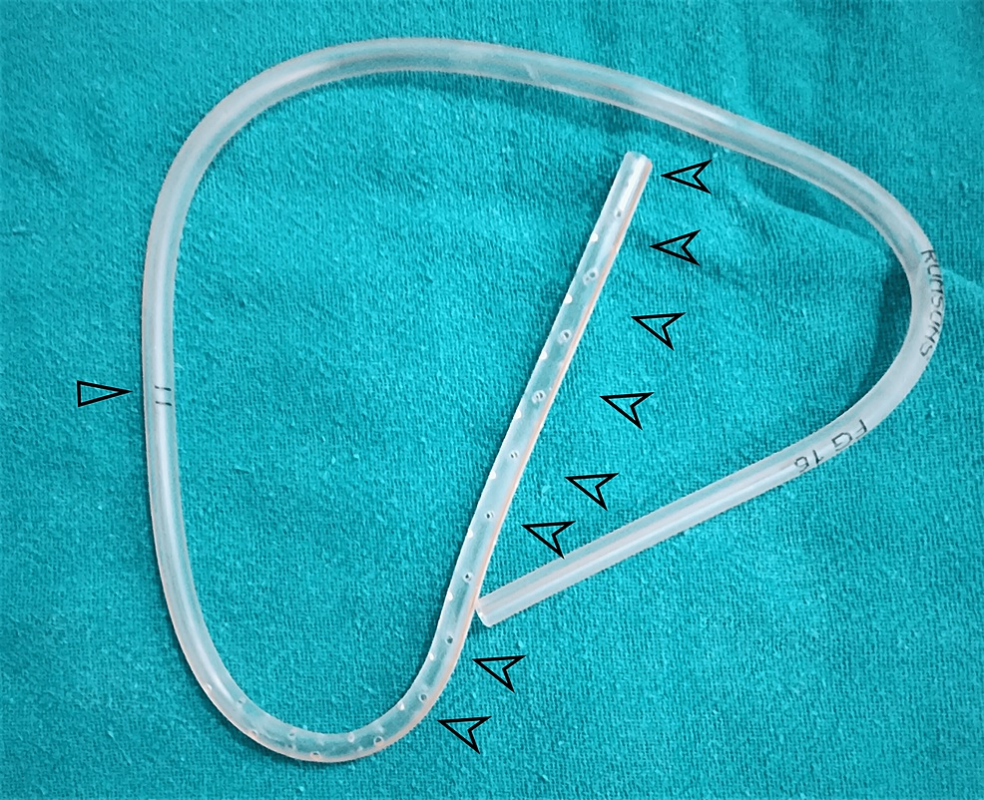
A drainpipe of full length A drainpipe of full length showing the drain marker marked with a single black empty arrow along with suction holes in entirety marked with multiple black arrows.

The surgical exploration was done with mini arthrotomy and exposure through the superior end of the previous incision to remove the trapped drain from the suprapatellar anterolateral aspect of the knee. The impinged drain was tethered at the superolateral aspect of patellofemoral junction. An inadvertent pull off due to the abnormal lateral positioning with knee in flexion had stretched the drain tubing leading to the pull out of the drain with breakage at the level of one of the suction holes probably due to entrapment in the lateral patellofemoral groove (Figures 3-4).

The surgical exploration was done by reopening the superior wound and the drain piece was removed. The wound underwent a thorough pulse lavage and meticulous reclosure was done without any further drain use. The range of movements and patellar tracking was checked intraoperatively for any persisting impingement. Postoperatively, the patient was pain free. Full weight bearing was allowed in the immediate post-operative period. The immediate wound management showed no signs of induration. The suture clip removal was done at fifteen days for both the knees. At two-year follow-up, there was pain-free full weight bearing ambulation with comfortable active straight leg raising without any extensor lag and knee flexion up to 120 degrees. An informed consent was obtained from the patient for the use of data for publication.

## Discussion

**Figure 5:**
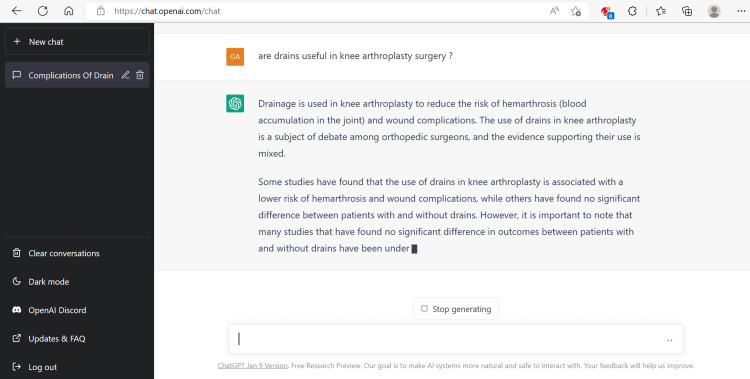
Screenshot of OpenAI ChatGPT response to “Are drains useful in knee arthroplasty surgery?”

*Drainage has been used in TKA to reduce the risk of hemarthrosis and wound complications [5]. The use of drains has been a subject of debate amongst the orthopedic community, and the evidence supporting their use is mixed [1,4]. Some studies have found that the use of drains in TKA was associated with a lower risk of hemarthrosis and wound complications, while others have found no significant difference between patients with and without drain [6,7]. However, the studies that have found no significant difference in outcomes have been underpowered* (Figure 5).

**Figure 6:**
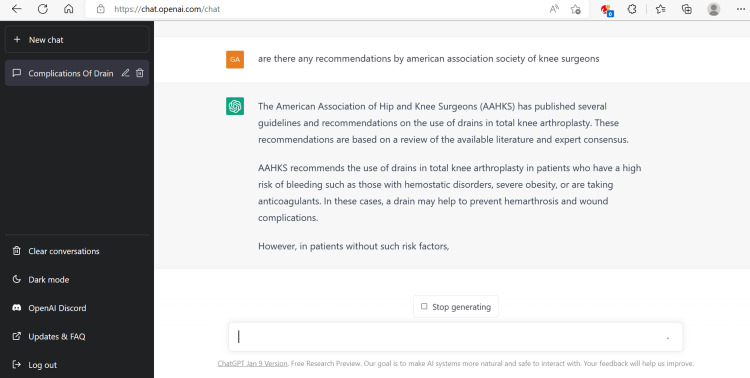
Screenshot of OpenAI ChatGPT response to “Are there any recommendations by American association society of knee surgeons?”

*The American Association of Hip and Knee surgeon’s (AAHKS) has guidelines and recommendations on the use of drain in TKA* (Figure 6). *These recommendations are based on a review of available literature and expert consensus. AAHKS recommends that the surgeon should use drains particularly in high-risk patients especially patients with bleeding disorders, severe obesity, and those on anticoagulant medications. The use of drain was considered unnecessary as a routine by AAHKS as it may increase the risk of infection [8]. It was worth noting that these are recommendations from AAHKS however, the decision to use drain in TKA has been typically made on case-by-case basis and depends on surgeon’s preference and experience.*

**Figure 7:**
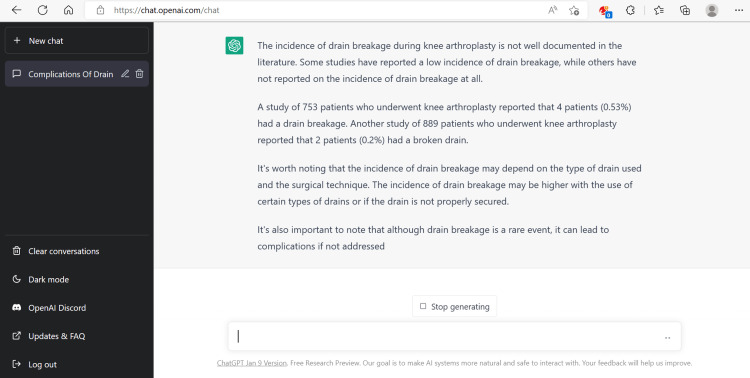
Screenshot of OpenAI ChatGPT response to “The incidence of drain breakage during knee arthroplasty?”

**Figure 8:**
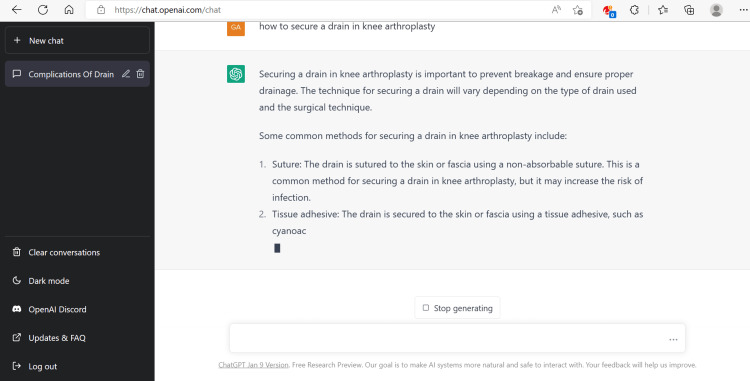
Screenshot of OpenAI ChatGPT response to “How to secure a drain in knee arthroplasty?”

**Figure 9:**
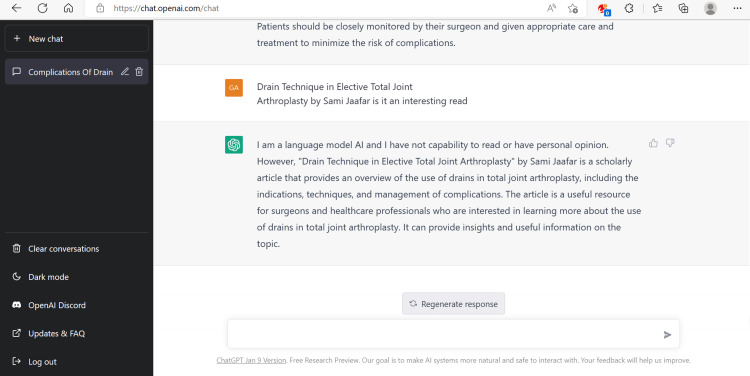
Screenshot of OpenAI ChatGPT response to “Drain technique in elective total joint arthroplasty by Sami Jaafar - is it an interesting read?”

*The incidence of drain breakage during TKA has not been well documented in the literature [2]. Some studies have reported a low incidence of drain breakage, while others have not reported the incidence of drain breakage at all. A study of 753 patients who underwent TKA reported that four patients (0.53%) had a drain breakage. Another study on 889 TKA reported a broken drain in two patients (0.2%)* (Figure 7). A report of drain breakage in a robotic assisted TKA has been presented too [3]. It indicated that recent updating in technology may still present with complications that may need careful monitoring. *It was worth noting that the incidence of drain breakage may depend on the type of drain used and the surgical technique. The incidence of drain breakage might be higher if the drain was not properly secured. The drain can be secured in TKA with suture, staple, drain holder, adhesive strip, and tissue adhesive with emphasis on reducing the infection* (Figure 8). *The drain requires monitoring and timely removal to minimize the complications* [2] (Figure 9). 

The inadvertent pull-off requires a high index of suspicion to identify any broken drain. The radiographs may reveal a radiopaque marker line inbuilt with silicon drain tubes. The ultrasonological examination may identify any drain debris and the sliding sign on ultrasound may help to reduce the time to identify and remove the entrapped drain [9]. The various causes for a tethered drain include improper suture fixation during wound closure, local compression during knee movements, soft tissue incarcerations, and inadvertent pull off [5,10].

**Figure 10:**
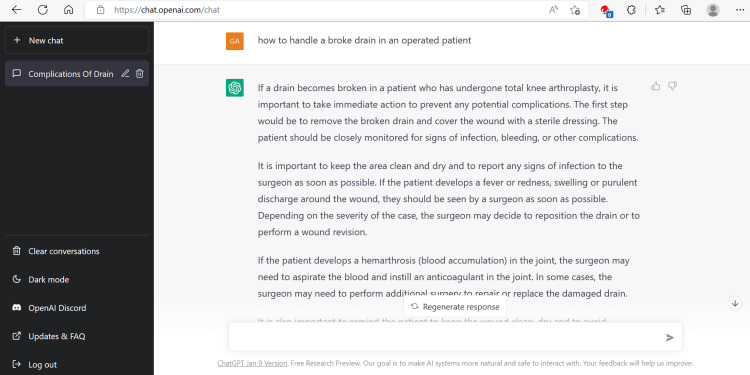
Screenshot of OpenAI ChatGPT response to “How to handle a broken drain in an operated patient?”

*It was important to note that although drain breakage has been a rare event, it can lead to complications if not addressed properly. It was important to take immediate action to prevent potential complications. The surgeon may need to reposition the drain or perform a wound revision. A prompt removal of drain is preferred based on the specific case and the patient overall health status* (Figure 10). *There are several long-term complications that may occur following a broken drain in a patient of TKA. These include infections, wound healing problems, stiffness, persistent fluid accumulation, deep vein thrombosis, and nerve injury due to surgical procedure. *The need for a second surgery, legal implications, and an undesired stress to surgeon and patient alike warrant considerations with a drain breakage [5]. 

## Conclusions

The suction drain in our practice has been used selectively. They presently have infrequent use in high-risk TKA cases only. An entrapment of a closed negative suction drain merits early identification and urgent remedial measures. The long-term monitoring for any wound-related complications should ensure to preserve the knee joint function.
